# Quercetin reverses β_1_-adrenoceptor autoantibody-induced heart failure by promoting MDM2-mediated ubiquitination and degradation of *p53* in cardiomyocytes

**DOI:** 10.3389/fnut.2025.1674507

**Published:** 2025-10-16

**Authors:** Mingxia Ma, Xintai Jiang, Weiqian Liu, Jin Xue, Yuan Yuan, Xiaoyan Zhi, Jiayan Feng, Yaolin Long, Yang Li, Zhijun Zhang, Xiaohui Wang, Li Wang

**Affiliations:** ^1^Department of Pathology, Shanxi Medical University, Taiyuan, Shanxi, China; ^2^Morphology Laboratory, Shanxi Medical University, Taiyuan, Shanxi, China; ^3^Department of Cardiology, Shanxi Bethune Hospital, Shanxi Academy of Medical Sciences, Third Hospital of Shanxi Medical University, Tongji Shanxi Hospital, Taiyuan, China

**Keywords:** heart failure, β_1_-adrenoceptor autoantibody, autophagy, ubiquitination, quercetin

## Abstract

**Introduction:**

Cardiomyocyte autophagy is essential for preserving cardiac homeostasis. Previous studies revealed that β_1_-adrenergic receptor autoantibody (β_1_-AA) suppressed cardiomyocyte autophagy, triggering cell death and heart failure (HF). Qiliqiangxin capsule enhances autophagy and mitigates HF through multiple pathways, but its complex composition complicates mechanistic clarity. Network pharmacology identified quercetin as a pivotal autophagy-inducing component in Qiliqiangxin, yet its role in counteracting β_1_-AA-induced autophagy impairment remains unvalidated. In this study, quercetin’s therapeutic potential and mechanisms in restoring autophagy in β_1_-AA-associated HF were investigated.

**Methods:**

Bioinformatics methods, including a STRING database analysis, PPI network construction, and Cytoscape-based pathway mapping, were used to delineate quercetin’s autophagy-related targets. The *in vivo* efficacy was assessed in β_1_-AA-positive mice treated with quercetin (100 mg/[kg·d], intraperitoneal). The *in vitro* validation used H9c2 cardiomyocytes pretreated with quercetin (100 μM) prior to β_1_-AA exposure. Autophagy markers, *p53* signaling, and ubiquitination pathways were analyzed by immunoblotting and functional enrichment analysis using the GOrilla database. A *p53* knockdown and overexpressing cardiomyocyte model confirmed pathway specificity.

**Results:**

Quercetin administration significantly restored myocardial autophagy levels in β_1_-AA-positive mice, which improved cardiac function and survival rates. In H9c2 cells, quercetin pretreatment reversed β_1_-AA-induced autophagy suppression. Bioinformatics linked quercetin to *p53* pathway modulation, with experimental validation showing quercetin downregulated *p53* expression *via* MDM2-mediated ubiquitination. *p53* knockdown enhanced autophagy, while its overexpression blocked quercetin’s effect, indicating quercetin restores autophagy in a *p53*-dependent manner. GO enrichment highlighted the association between quercetin and ubiquitin-dependent protein degradation, which was corroborated by elevated MDM2 levels and accelerated *p53* degradation in quercetin-treated cells.

**Discussion:**

Quercetin rescues β_1_-AA-impaired cardiomyocyte autophagy by activating MDM2-dependent *p53* ubiquitination and degradation, thereby attenuating HF progression. These findings establish quercetin as the mechanistic basis of the cardioprotective effects of Qiliqiangxin and provide preclinical evidence for targeting autophagy by regulating *p53* in β_1_-AA-induced cardiac dysfunction.

## 1 Introduction

Heart failure, often the terminal stage of various cardiovascular diseases, continues to be a major clinical challenge despite significant progress in therapeutic approaches. Even with treatment, a considerable proportion of patients still suffer from persistent symptoms, with high rates of hospital re-admission and mortality due to disease progression (1). Therefore, identifying targeted pharmacological therapies for heart failure (HF) remains a critical need in the field of cardiovascular research.

Our previous research found that β_1_-adrenergic receptor autoantibody (β_1_-AA) inhibited autophagy levels in cardiomyocytes, leading to HF (2). β_1_-AA is an autoantibody directed against the second extracellular loop (ECII) of the β_1_-adrenergic receptor (β_1_-AR). Clinical investigations have reported its presence in the serum of approximately 40–60% of patients with HF (3). Removing β_1_-AA from patients’ blood using immunoadsorption has significantly improved cardiac function, suggesting a close relationship between β_1_-AA and HF (4, 5). Although rapamycin-induced upregulation of autophagy levels in cardiomyocytes has effectively improved β_1_-AA-induced myocardial injury and dysfunction (2), the clinical use of rapamycin as an immunosuppressant is associated with significant side effects, including the risk of severe infections and death (6). Therefore, identifying safe and effective drugs to upregulate cardiomyocyte autophagy is crucial for improving β_1_-AA-induced HF.

An increasing amount of evidence suggests that traditional Chinese medicine is both safe and effective as a HF treatment (7). The Chinese guidelines for chronic HF treatment highlight Qiliqiangxin capsule as an effective therapy for HF (8, 9). Qiliqiangxin capsule is a compound herbal medicine consisting of 11 ingredient plants: *Panax ginseng*, *Astragalus*, *Rhizoma aconiti lateralis radix preparata*, *Alisma*, *Salvia miltiorrhiza*, *Carthami flos*, *Semen descurainiae lepidii*, *Cinnamomi ramulus*, *Citri reticulatae pericarpium*, *Polygonati odorati rhizome*, and *Cortex Periplocae* (10). Some studies reported that Qiliqiangxin capsule exhibited protective effects on the heart in HF patients (9, 11, 12), but the specific mechanisms are not clear. In this study, quercetin was identified through network pharmacology analysis as the key component of Qiliqiangxin capsule promoting autophagy. Quercetin is present in multiple herbal components of Qiliqiangxin capsules, with *Astragalus*, *Carthami flos* and *Semen descurainiae lepidii* being the main botanical sources. As a flavonoid that is widely distributed in nature, quercetin enhances mitophagy *via* the SIRT1/TMBIM6 pathway, thereby improving mitochondrial function and protecting cardiomyocytes (12). Quercetin was also found to regulate miR-223-3p/FOXO3 axis to promote autophagy and suppress isoproterenol-induced myocardial fibrosis (13). Despite promising indications, the potential of quercetin to reverse the β_1_-AA-induced autophagy decline and its underlying mechanisms remain unclear. This study combined bioinformatics and molecular biology methods to investigate the molecular mechanisms by which quercetin reverses β_1_-AA-induced autophagy suppression and improves HF in mice, providing experimental evidence for the therapeutic potential of quercetin in autophagy-related cardiovascular diseases.

## 2 Materials and methods

### 2.1 Data collection and processing

Microarray data for HF were obtained from the Gene Expression Omnibus (GEO) database (including GSE57338, GSE5406 and GSE3586). The datasets were merged using the R package in SilicoMerging (14), and batch effects were corrected with the Empirical Bayes method proposed by Johnson et al. (15). A differential expression analysis of the data was conducted using the limma R package (version 3.40.6) (16) to identify differentially expressed genes (DEGs). A gene set enrichment analysis of the DEGs was performed using the R package clusterProfiler (version 3.14.3) (17).

### 2.2 Screening of active ingredients in Qiliqiangxin capsule

The active ingredient candidate components of Qiliqiangxin capsule were obtained from the TCMSP,^1^ which is a valuable tool for investigating the pharmacological mechanisms underlying traditional Chinese medicine (18). Oral bioavailability (OB) refers to the fraction of a drug that reaches systemic circulation, reflecting the absorption rate of active pharmaceutical ingredients. Pharmacokinetic studies are used to determine OB, with higher values indicating better absorption (19). Druglikeness (DL) assesses a small molecule’s potential as an orally bioavailable drug. Researchers have suggested that molecules with OB ≥ 30% or DL ≥ 0.18 have enhanced pharmacological effects (20). Therefore, in this study, the screening criteria for active ingredients in Qiliqiangxin capsule were defined as compounds meeting both OB ≥ 30% and DL ≥ 0.18 thresholds.

### 2.3 Screening of target genes for HF

Target genes associated with HF were downloaded from the TTD^2^ (21), DisGeNET^3^ (22), and DrugBank^4^ (23) databases. The datasets from these three databases were integrated by taking their union to obtain a consolidated set of HF-related target genes.

### 2.4 Construction of the HF-target gene-active ingredient network

To elucidate the multi-scale relationships among the active ingredients of Qiliqiangxin capsule, HF-related targets and autophagy-related targets, we constructed an HF-target gene-active ingredient network. The active ingredients and their corresponding targets were derived from the screening process described in Section 2.2, while the HF-related genes were sourced from the databases listed in Section 2.3. A Venn diagram was used to identify the intersection of three specific gene sets: the targets of the active ingredients of Qiliqiangxin capsule, HF-related genes and autophagy-related genes. These overlapping genes were considered potential key targets through which Qiliqiangxin capsule exerts its therapeutic effects on HF by regulating autophagy. The tripartite network was visualized using Cytoscape (v3.7.2) (24). The network consists of nodes representing active ingredients, overlapping target genes, and HF, connected by edges that depict their interactions.

### 2.5 Construction of protein–protein interaction network

We conducted a protein–protein interaction (PPI) analysis using the STRING database,^5^ setting the species to “*Homo sapiens*” and applying a score threshold of >0.4 (25). The MCC method of the CytoHubba plugin in Cytoscape (24) was used to identify 10 key targets within the PPI network.

### 2.6 Molecular docking simulation

The MDM2 protein structure was retrieved from the RCSB PDB database^6^ (26), while the molecular structure of quercetin was sourced from the Traditional Chinese Medicine Systems Pharmacology Database (TCMSP) platform. Before docking with AutoDockTools (27), we protonated the molecules and assessed their charge states. Docking was performed using Vina with optimized docking site settings. The results were visualized using PyMOL (28).

### 2.7 Molecular dynamics simulations

The molecular dynamics simulations were conducted using the academic version of Desmond/Maestro (v2022.1). The simulation systems were solvated using a TIP3P water model and neutralized with 0.15 M sodium chloride solution. Following energy minimization and system equilibration, a 100-ns production run was executed under NPT conditions (300 K, 1 atm). Trajectory snapshots were captured at 100-ps intervals for subsequent analysis. All post-simulation analyses were carried out using a built-in simulation interaction diagram tool in Desmond.

### 2.8 Animal models

Male C57BL/6 mice 6–8 weeks of age were obtained from the Experimental Animal Center of Shanxi Medical University. An active immunization model was generated based on protocols outlined in earlier studies (2, 29). The quercetin monotherapy group and the quercetin treatment group were each administered quercetin (Q4951, Sigma Aldrich) via intraperitoneal injection at a dose of 100 mg/(kg· d) (30) for 4 consecutive weeks after the initial primary immunization. The mice were anesthetized via intraperitoneal injection of sodium pentobarbital at a concentration of 1% in saline, administered at a dose of 40 mg/kg body weight. Blood was drawn from the retro-orbital venous plexus of the mice and centrifuged at 3,000 × g for 10 min. Serum β_1_-AA concentrations in actively immunized mice were subsequently determined using the SA-ELISA (2, 29, 31) (Supplementary Figure S1). At the end of the experiment, the mice were euthanized by intraperitoneal injection of an overdose of sodium pentobarbital, with death confirmed by cessation of breathing and heartbeat. All animal experiments were performed in strict compliance with the ARRIVE guidelines and relevant regulations, with protocols approved by the Experimental Animal Ethics Committee of Shanxi Medical University (Approval No. SYDL2024037).

### 2.9 Small animal ultrasound

The echocardiographic analysis primarily included parameters such as left ventricular internal systolic diameter (LVIDs), left ventricular internal diastolic diameter (LVIDd), end-systolic volume (ESV), and end-diastolic volume (EDV), along with reduced ejection fraction (EF) and fractional shortening (FS).

### 2.10 Masson staining

After fixation and dehydration, the cardiac tissue was embedded in paraffin and cut into 5 μm sections. Masson staining was performed to examine the histological morphology of the cardiac tissue.

### 2.11 Cell culture and treatments

H9c2 cells (GNR 5) were cultured as described in our previous studies (2, 29). The knockdown efficiency was verified, with the results presented in Supplementary Figure S2. Stable knockdown clones were selected for subsequent stimulation experiments. The following cell groups were included in the study: the control group (treated with 1 μM negative IgG for 24 h), the quercetin group (pretreated with 100 μM quercetin for 4 h), the β_1_-AA group (treated with 1 μM β_1_-AA for 24 h), the β_1_-AA + quercetin group (pretreated with 100 μM quercetin for 4 h, then treated with 1 μM β_1_-AA for 24 h), and the *p53* knockdown+β_1_-AA + quercetin group (In the stably transfected *p53* knockdown H9c2 cells, pretreatment with 100 μM quercetin was performed for 4 h, followed by treatment with 1 μM β_1_-AA for 24 h).

### 2.12 RNA preparation and RT-Q-PCR

Quantitative PCR was performed using the RT-qPCR rapid qPCR hybrid kit containing TB Green (Takara, RR430A, JPN). The primer sequences can be found in Supplementary Table S1.

### 2.13 Co-immunoprecipitation

Protein A/G magnetic beads were incubated with *p53* antibody and rabbit IgG antibody at 4 °C for 6 h, followed by an overnight incubation with protein lysates. The protein was eluted from the beads and denatured before being analyzed by western blotting.

### 2.14 Western blot

Western blot analyses were performed as previously described (29). The antibody catalog numbers are listed in Supplementary Table S2.

### 2.15 Immunofluorescence

Cells were fixed on slides with a 1:1 mixture of methanol and propylene glycol for 15 min, followed by three washes. The cells were blocked with a 5% bovine serum albumin (BSA) solution for 1 h. Next, the cells were incubated with *p53* antibody at 4 °C for 16 h, and then with fluorescent secondary antibody at 37 °C for 1 h in the dark. Finally, the slides were mounted with a medium containing DAPI, and immunofluorescence images were captured using a confocal laser scanning microscope.

### 2.16 Plasmid transfection and detection

Sterile cell climbing slices were pre-placed in 12-well plates, and H9c2 cells were seeded at an appropriate density onto the slices and cultured for 24 h. Transfection of GFP-LC3 plasmid was carried out using Lip3.0 reagent (Hanbio Co., Ltd., Shanghai, China) as follows: 4 μg of plasmid was added to 250 μL of DMEM in a sterile tube and gently mixed; separately, 6 μL of LipoFiter3.0 reagent (Hanbio) was mixed with 250 μL of Dulbecco’s Modified Eagle Medium (DMEM) in another sterile tube and incubated at room temperature for 5 min. The DNA solution was then combined with the LipoFiter3.0 solution, mixed gently, and incubated at room temperature for 20 min to form complexes. The resulting complexes were added dropwise to the plates containing cell climbing slices. After 6 h of transfection, the medium was replaced with complete medium containing 10% fetal bovine serum (2 mL). At 48 h post-transfection, the cell climbing slices were collected, washed with Phosphate-Buffered Saline (PBS), fixed with 4% paraformaldehyde for 15 min, and permeabilized with 0.1% Triton X-100 for 10 min. The slices were then incubated with 4′,6-diamidino-2-phenylindole (DAPI) staining working solution in the dark for 10 min to label nuclei. After washing three times with PBS, the slices were mounted with antifade mounting medium and observed under a laser scanning confocal microscope to quantify GFP-LC3 puncta and assess nuclear morphology.

### 2.17 Statistical analysis

Student’s *t*-test was used to compare differences between two groups, and one-way ANOVA with Bonferroni *post hoc* test was used for multiple group comparisons. *p* < 0.05 was considered statistically significant.

## 3 Results

### 3.1 GEO database analysis revealed significant enrichment of autophagy-related pathways in the myocardial tissue of HF patients

**Figure 1:**
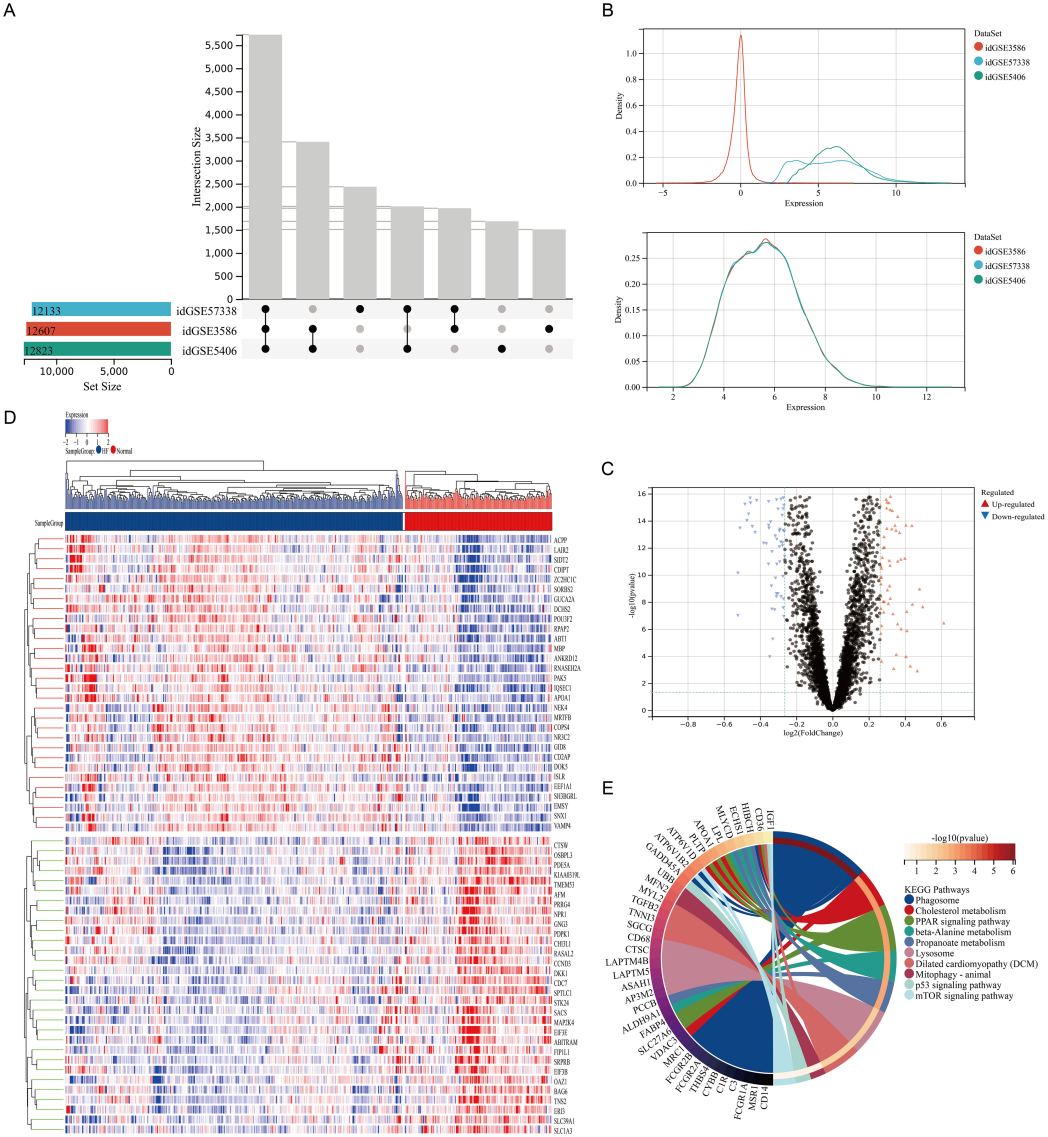
GEO database analysis revealed significant enrichment of autophagy-related pathways in the myocardial tissue of HF patients. **(A)** UpSet plot analysis of three HF datasets. Left bars (X: Set Size) show the total genes per dataset. The main plot (Y: Intersection Size) shows genes shared by specific dataset combinations, with dots representing unique gene counts for the corresponding connected sets below, identifying common and unique genes. **(B)** Batch effect correction using an empirical Bayes method. Density distributions of gene expression show multimodal patterns before correction and unimodal patterns after correction (X, gene expression values; Y, probability density). **(C)** Volcano Plot of DEGs. The plot shows gene expression changes [X: log_2_FC; Y: −log_10_(adj. *P*-value)]. Red, significantly upregulated genes (*p* < 0.05, log_2_FC ≥ 0.263); blue, significantly downregulated genes (*p* < 0.05, log_2_FC ≤ −0.263); gray, non-significantly regulated genes. **(D)** Heatmap of DEGs showing the expression of significantly differentially expressed genes (adj. *p* < 0.05, |log_2_FC| ≥ 0.263) across samples. Rows, genes; columns, samples by group. Color gradient, blue (low) to red (high) expression (row Z-score normalized). **(E)** KEGG enrichment chord plot of DEGs, showing significant pathway-gene interactions (adj. *p* < 0.05). Outer arc, pathway terms; inner arc, DEGs. Ribbon width indicates enrichment strength; color corresponds to −log_10_(adj. *p*-value).

To systematically investigate key pathway alterations in myocardial tissue in the pathogenesis of HF, we integrated three gene expression microarray datasets (GSE5406, GSE57338, GSE3586) derived from human myocardial tissue (Table 1). After batch effect correction and merging the datasets (Figures 1A,B), a cohort consisting of 385 HF patients and 164 healthy controls was constructed. An analysis of the differential expression identified a total of 213 significantly DEGs, with 109 significantly upregulated and 104 significantly downregulated (Figures 1C,D; Supplementary Table S3). To further elucidate the biological functions of these DEGs, a KEGG pathway enrichment analysis was performed. The results demonstrated significant enrichment of autophagy-related pathways, including phagosome, lysosome, mitophagy-animal, and mTOR signaling pathways (Figure 1E). These findings suggested that autophagy dysregulation in myocardial tissue may contribute to human HF.

**Table 1:** The number of sample for HF and normals in include datasets.

Tissue	GSE	platform	HF	Healthy
	GSE57338	Gpl11532	177	136
Lelf ventricle	GSE5406	Gpl125134	193	16
	GSE3586	Gpl1153296	15	12

Sample distribution of HF and Healthy tissues across included datasets from the GEO. The table summarizes the number of samples for each condition (HF and Normal) along with the corresponding GSE accession numbers and platform identifiers for left ventricular cardiac tissue.

### 3.2 Quercetin is a key active component in Qiliqiangxin capsule that upregulates autophagy

**Figure 2:**
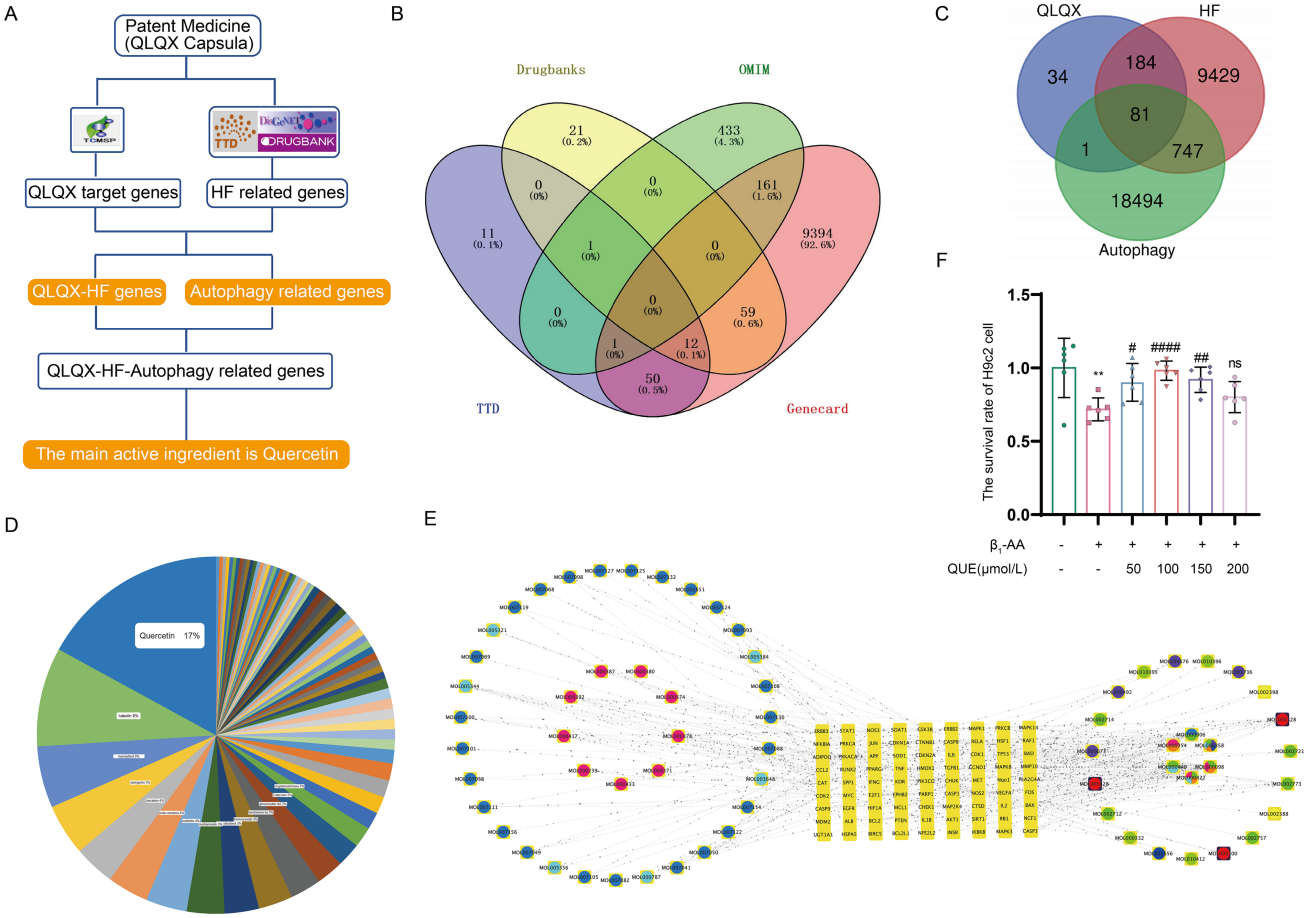
Quercetin is a key active component in Qiliqiangxin capsule autophagy upregulation. **(A)** Network pharmacology workflow diagram. **(B)** Integration of HF genes from DrugBank, OMIM, TTD, and GeneCards. **(C)** The intersection of Qiliqiangxin capsule HF-related targets with autophagy-related genes identified in 81 autophagy-related genes. **(D)** Chart indicating the proportions of active components in Qiliqiangxin capsule related to HF and autophagy, with quercetin having the highest proportion. **(E)** Network diagram of traditional Chinese medicine (TCM) active components in Qiliqiangxin capsule related to HF and autophagy. Yellow square nodes represent target genes; red, yellow, green, purple and blue nodes represent TCM herbs; circular nodes represent active components. **(F)** Effect of quercetin on β_1_-AA-induced H9c2 cardiomyocyte death. Quercetin was tested at concentrations of 50, 100, 150, and 200 μM. The CCK8 assay showed that 100 μM quercetin significantly attenuated β_1_-AA-induced H9c2 cardiomyocyte death (^**^p < 0.01 vs. Ctrl; ^#^*p* < 0.05, ^##^*p* < 0.01, ^####^*p* < 0.0001 vs. β_1_-AA group, *n* = 6).

In this research, a network pharmacology analysis was performed to identify the key constituents in the traditional Chinese formulation, Qiliqiangxin capsule, that are responsible for enhancing autophagy (Figure 2A). We used the TCMSP database to obtain the active components and corresponding protein targets (Supplementary Table S4) of the 11 herbs in Qiliqiangxin capsule (*Panax ginseng*, *Astragalus*, *Rhizoma aconiti lateralis radix preparata*, *Alisma*, *Salvia miltiorrhiza*, *Carthami flos*, *Semen descurainiae lepidii*, *Cinnamomi ramulus*, *Citri reticulatae pericarpium*, *Polygonati odorati rhizome*, and *Cortex Periplocae*). By searching multiple online databases (Disgenet, GeneCards, TTD, and DrugBank), we identified 265 targets related to HF treatment (Figure 2B) from Qiliqiangxin capsule (Supplementary Table S5). To find the targets related to both Qiliqiangxin capsule and autophagy, we intersected the 265 HF-related targets with autophagy-related genes (Figure 2C), which identified 81 autophagy-related genes (Supplementary Table S6). Further analysis of the active components corresponding to these 81 autophagy-related genes revealed that quercetin had the highest proportion (Figures 2D,E). We then explored how quercetin influences cardiomyocyte death mediated by β_1_-AA. We tested quercetin at four concentrations: 50, 100, 150, and 200 μM. The results of a CCK8 assay indicated that quercetin at concentrations of 50, 100, and 150 μM mitigated β_1_-AA-induced death of H9c2 cardiomyocytes, with the 100 μM concentration demonstrating a significant protective effect against β_1_-AA-induced H9c2 cardiomyocyte death (Figure 2F).

### 3.3 Quercetin reverses β_1_-AA-induced myocardial fibrosis and cardiac dysfunction in mice

**Figure 3:**
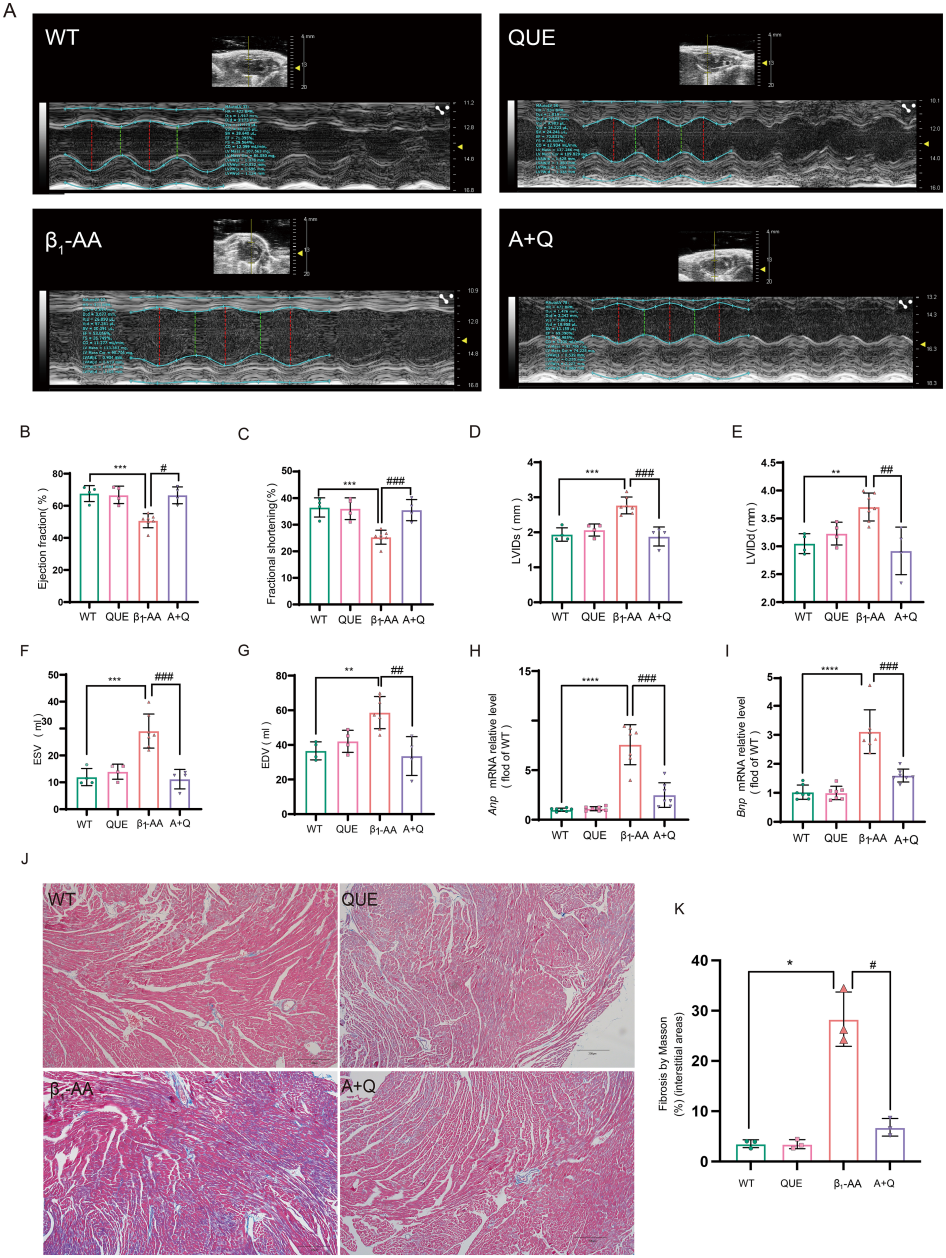
Quercetin reverses β_1_-AA-induced cardiac pathological and functional abnormalities in mice. **(A–G)** Evaluation of EF%, FS%, LVIDd, LVIDs, ESV, and EDV using small animal ultrasound (*n* = 4–6). **(H,I)** Quantitative analysis of atrial natriuretic peptide (*Anp*, *n* = 6) and brain natriuretic peptide (*Bnp*, *n* = 7) mRNA expression in myocardial tissue. **(J)** Collagen accumulation assessed via Masson’s trichrome staining, with collagen fibers indicated in blue. **(K)** Results of the quantitative analysis of collagen deposition (*n* = 3). Values are expressed as mean ± SD; ^*^p < 0.05, ^**^p < 0.01, ^***^p < 0.001, ^****^p < 0.0001 vs. WT; ^#^*p* < 0.05, ^##^*p* < 0.01, ^###^*p* < 0.001 vs. β_1_-AA group.

To evaluate the therapeutic effect of quercetin on β_1_-AA-induced HF, an HF phenotype was established in mice through active immunization with β_1_-AR-ECII peptide *via* subcutaneous multi-point injections over a four-week period, with booster immunizations administered every 2 weeks. Concurrently with the modeling, the treatment group received daily intraperitoneal injections of 100 mg/kg quercetin to assess its therapeutic efficacy. To determine the effects of quercetin on cardiac function, we performed echocardiography. We found that the β_1_-AA group of mice developed significant cardiac dysfunction, specifically manifested as increased LVIDs, LVIDd, ESV, and EDV, along with reduced EF and FS. Quercetin treatment significantly reversed these abnormal cardiac function indicators (Figures 3A–G), indicating that quercetin exerted a protective effect on cardiac function. We next evaluated myocardial histopathological changes by analyzing the degree of myocardial fibrosis using Masson staining. The results showed significant collagen deposition in the myocardial interstitium of mice in the β_1_-AA group, while quercetin treatment markedly attenuated this pathological fibrotic change (Figures 3J,K). Finally, we measured the mRNA expression of the HF markers *Anp* and *Bnp*. β_1_-AA significantly upregulated the expression of these two markers, further confirming impaired cardiac function in the model group, whereas quercetin treatment effectively suppressed the increased expression of these markers (Figures 3H,I).

### 3.4 Quercetin reverses β_1_-AA-induced decrease in myocardial cell autophagy

**Figure 4:**
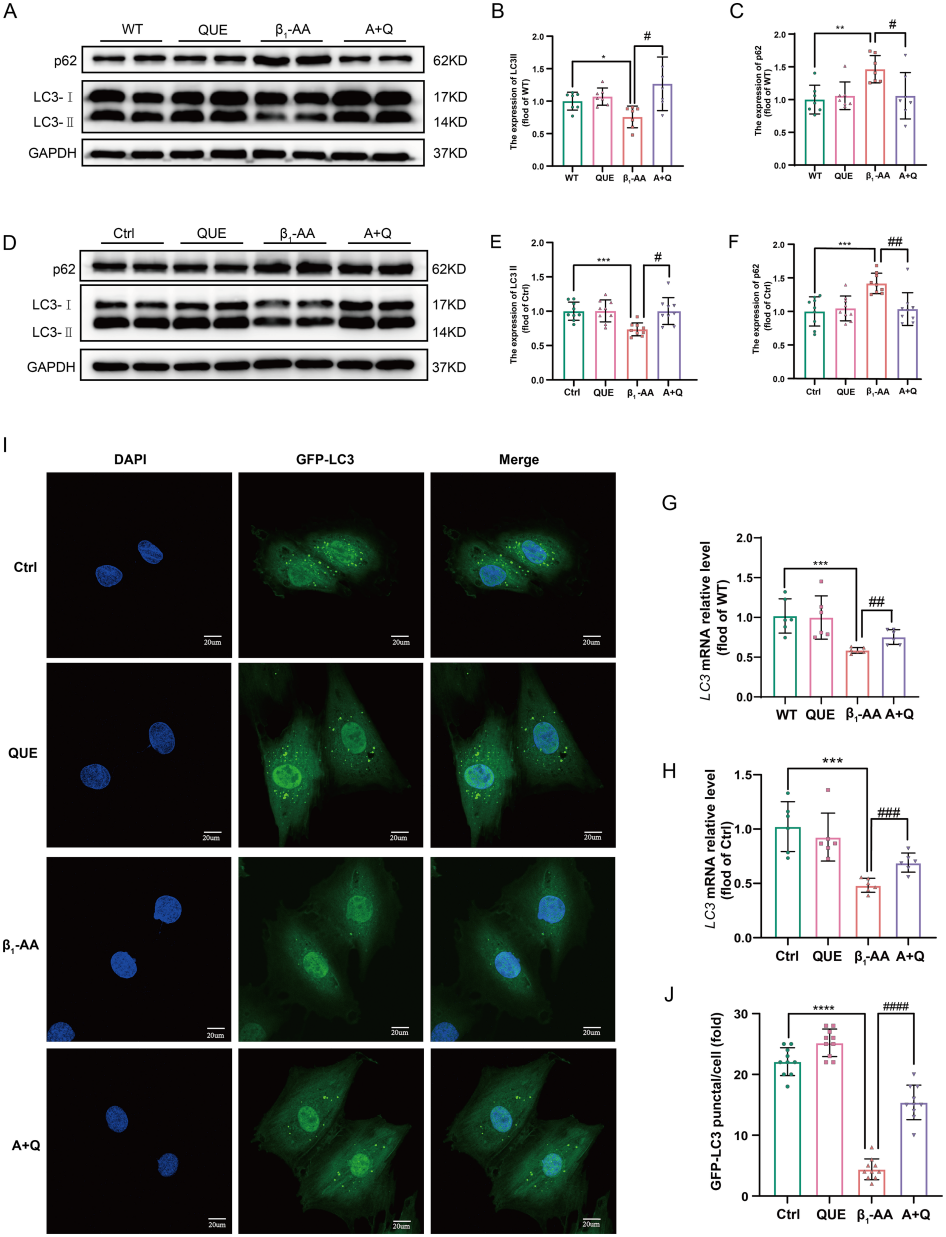
Quercetin reverses the decrease in myocardial cell autophagy induced by β_1_-AA. **(A)** Western blot analysis was performed to assess LC3 and p62 protein levels in C57BL/6 mice actively immunized with the β_1_-AR-ECII peptide and treated with quercetin for 4 weeks (*n* = 7). **(D)** Western blot analysis of LC3 and p62 protein levels in H9c2 cardiomyocytes following 24 h of β_1_-AA exposure (*n* = 8). **(G)** *LC3* mRNA expression in mice actively immunized with the β_1_-AR-ECII peptide for 4 weeks and treated with quercetin, as determined by RT-PCR (*n* = 6). **(H)** *LC3* mRNA expression in H9c2 cells was measured after 24 h of β_1_-AA treatment (*n* = 6). **(I)** Immunofluorescence staining showing LC3 puncta (green fluorescence; scale bar = 20 μm). **(J)** Quantification of LC3 puncta number (*n* = 10). **(B,C,E,F)** Statistical charts for LC3 and p62. Data are expressed as mean ± SD, ^*^p < 0.05, ^**^p < 0.01, ^***^p < 0.001 vs. Ctrl or WT; ^#^ *p* < 0.05, ^##^ *p* < 0.01, ^###^ *p* < 0.001 vs. β_1_-AA group.

To investigate the role of autophagy in quercetin-mediated protection against β_1_-AA-induced myocardial injury, key indicators of autophagic flux were systematically evaluated in both *in vivo* and *in vitro* models. In the cardiac tissue from β_1_-AA-positive mice, intraperitoneal administration of quercetin significantly increased LC3-II protein and *LC3* mRNA levels compared to those of the β_1_-AA group without quercetin treatment. Quercetin treatment also reduced the accumulation of the autophagy substrate p62 (Figures 4A–C, G). In H9c2 cells, pretreatment with 100 μM quercetin for 4 h prior to 24 h β_1_-AA exposure increased LC3-II levels and decreased p62 levels (Figures 4D–F, H). An immunofluorescence analysis revealed a higher number of LC3 puncta in quercetin-pretreated cardiomyocytes following β_1_-AA challenge compared to the β_1_-AA alone group (Figures 4I,J). These results collectively indicate that quercetin enhances autophagic flux and mitigates β_1_-AA-induced impairment of autophagy.

### 3.5 Quercetin reverses the reduction in myocardial cell autophagy induced by β_1_-AA by decreasing *p53* protein expression

**Figure 5:**
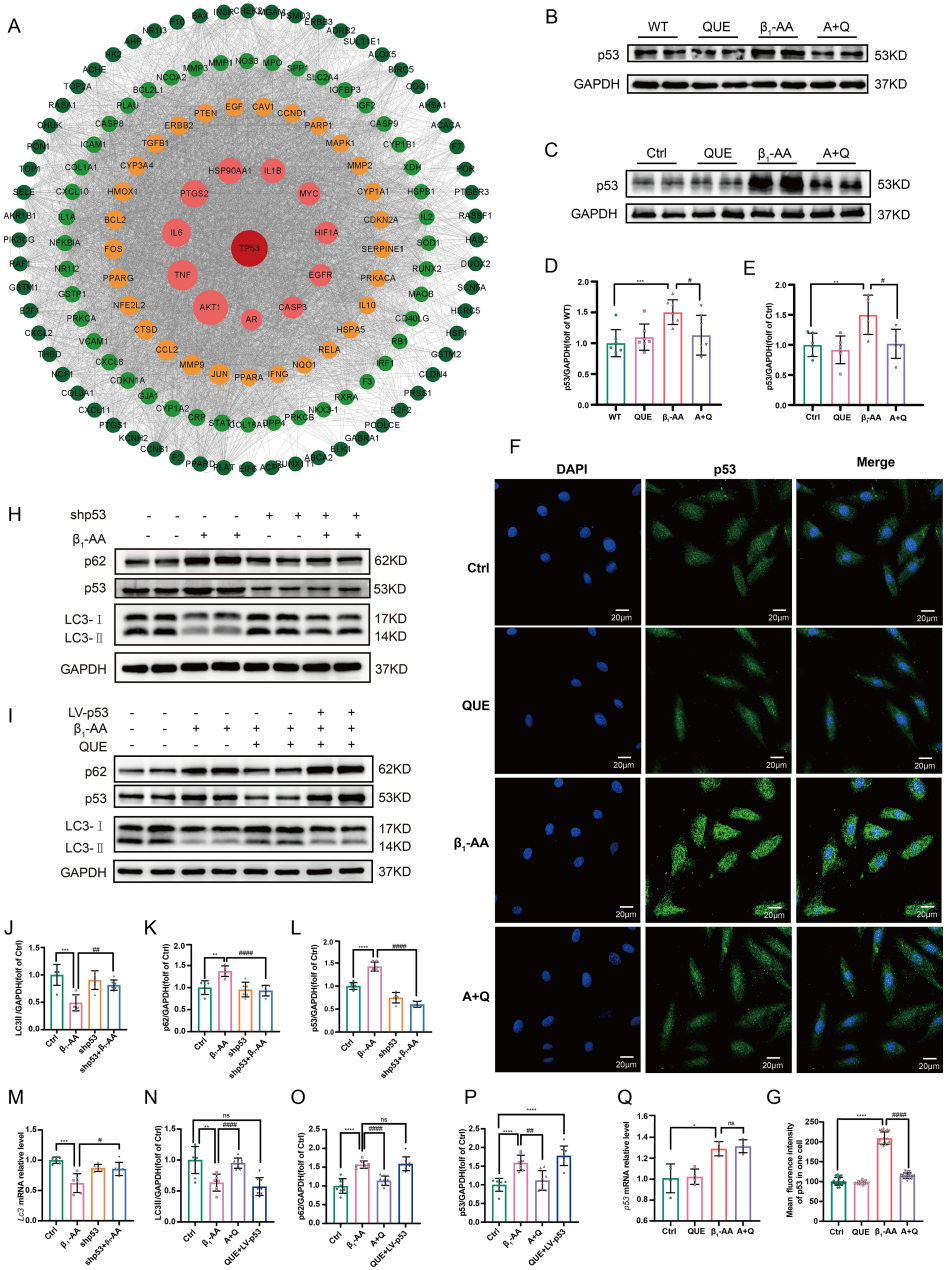
By decreasing *p53* protein expression, quercetin reversed the reduction in myocardial cell autophagy induced by β_1_-AA. **(A)** PPI network of quercetin target genes constructed using the STRING database and a regulatory network using Cytoscape. The cytoHubba algorithm identified *p53* as a hub gene. **(B)** Western blot analysis of *p53* protein levels in C57BL/6 mice actively immunized with β_1_-AR-ECII peptide segment for 4 weeks and treated with quercetin (*n* = 7). **(C)** Western blot analysis of *p53* protein levels in H9c2 cardiomyocytes after 24 h of β_1_-AA treatment (*n* = 6). **(D,E)** Statistical analysis of *p53* levels. **(F)** Immunofluorescence detection of *p53*. Green fluorescence represents *p53*, scale bar = 20 μm. **(G)** Quantification of *p53* immunofluorescence intensity. **(H,J,K,L)** Western blot analysis of the protein levels of LC3 and p62 in myocardial cells with lentiviral *p53* knockdown and quantified relative protein levels (*n* = 6). **(I,N,O,P)** Western blot analysis of LC3 and p62 protein levels in myocardial cells with *p53* overexpression mediated by a lentivirus, and quantification of the relative protein levels (*n* = 6). (M) RT-PCR analysis the *LC3* mRNA levels in myocardial cells with *p53* knockdown (*n* = 6). **(Q)** The expression level of *p53* mRNA in myocardial cells after quercetin treatment. Data are presented as means ± SD, ^*^p < 0.05, ^**^p < 0.01, ^***^p < 0.001, ^****^p < 0.0001 vs. Ctrl or WT; ^#^ *p* < 0.05, ^##^ *p* < 0.01 vs. β_1_-AA group; ns, not significant.

To explore the potential targets of quercetin, we used the STRING database to construct a PPI network based on its associated genes and visualized the regulatory network using Cytoscape. The cytoHubba algorithm identified *p53* as a key hub gene (Figure 5A). To validate the bioinformatic prediction, we examined *p53* protein expression using both *in vivo* and *in vitro* models. After 4 weeks of immunization, *p53* protein levels were significantly elevated in mouse myocardial tissues, while quercetin treatment markedly suppressed the β_1_-AA-induced upregulation of *p53* protein (Figures 5B,D) We further confirmed this regulatory relationship in a cell model, which produced consistent results (Figures 5C,E). An immunofluorescence analysis revealed a significant increase in the *p53* fluorescence intensity in β_1_-AA-treated cells, while quercetin treatment effectively reversed the β_1_-AA-induced enhanced signal (Figures 5F,G). We then performed lentivirus-mediated *p53* knockdown to investigate the role of *p53* in autophagy regulation. *p53* knockdown attenuated the β_1_-AA-induced decrease in both LC3-II protein and mRNA levels and reduced p62 accumulation (Figures 5H, J–M). To determine whether *p53* was essential to quercetin-induced autophagy, we established a *p53* overexpression model. In normal cells, quercetin treatment significantly restored autophagy levels; however, this restorative effect of quercetin was markedly attenuated in *p53*-overexpressing cells (Figures 5I, N–P). These results demonstrate that *p53* is indispensable for the regulation of autophagy by quercetin. Notably, while quercetin markedly reduced *p53* protein levels in cardiomyocytes, *p53* mRNA levels remained unchanged (Figure 5Q), indicating that quercetin may modulate *p53* post-transcriptional levels, possibly by promoting its protein degradation.

### 3.6 Quercetin reverses the decrease in *p53* ubiquitination levels induced by β_1_-AA in myocardial cells

**Figure 6:**
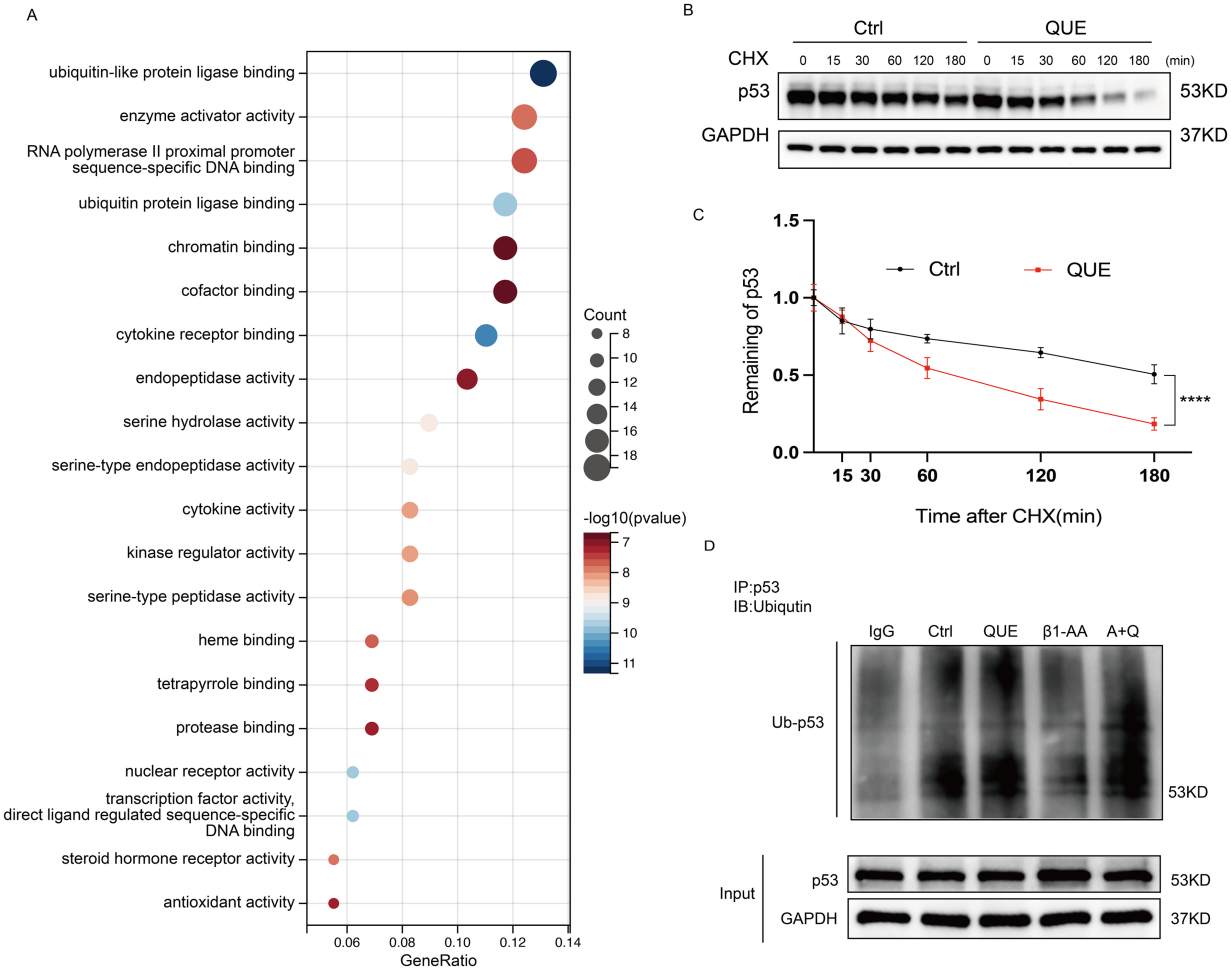
Quercetin reverses the decrease in *p53* ubiquitination levels induced by β_1_-AA in myocardial cells. **(A)** GO functional enrichment analysis of quercetin-targeted proteins using the R packages clusterProfiler, org. Hs.eg.db, enrichplot, and ggplot2. **(B)** H9c2 cardiomyocytes were pretreated with 100 μM quercetin for 4 h, followed by treatment with 10 μM CHX for the specified durations. Cells were analyzed by Western blotting using GAPDH as an internal control. **(C)** Quantitative analysis of protein expression levels (*n* = 4). Data are presented as mean ± SD. Statistical significance was determined by two-way ANOVA with Bonferroni’s multiple comparison test. ^****^p < 0.0001. **(D)** Co-immunoprecipitation (Co-IP) results show that compared to the control group, β_1_-AA treatment reduced *p53* ubiquitination levels in myocardial cells, which was significantly reversed by quercetin.

To investigate the role of quercetin in *p53* degradation, we performed GO functional enrichment analysis of quercetin-targeted proteins using R packages (clusterProfiler, org.Hs.eg.db, enrichplot, and ggplot2). The results revealed that quercetin-targeted proteins were significantly enriched in pathways related to ubiquitination (Figure 6A), with similar results obtained through GOrilla database analysis (Supplementary Figure S3). Additionally, H9c2 cardiomyocytes were pretreated with 100 μM quercetin for 4 h, followed by treatment with 10 μM cycloheximide (CHX) at specified time points. The results demonstrated that quercetin significantly increased the degradation rate of *p53* protein (Figures 6B,C). A further analysis of *p53* ubiquitination levels indicated that the reduced *p53* ubiquitination levels induced by *β*_1_-AA in cardiomyocytes were reversed by quercetin (Figure 6D).

### 3.7 Quercetin reverses the downregulation of MDM2 protein levels in myocardial cells induced by β_1_-AA

**Figure 7:**
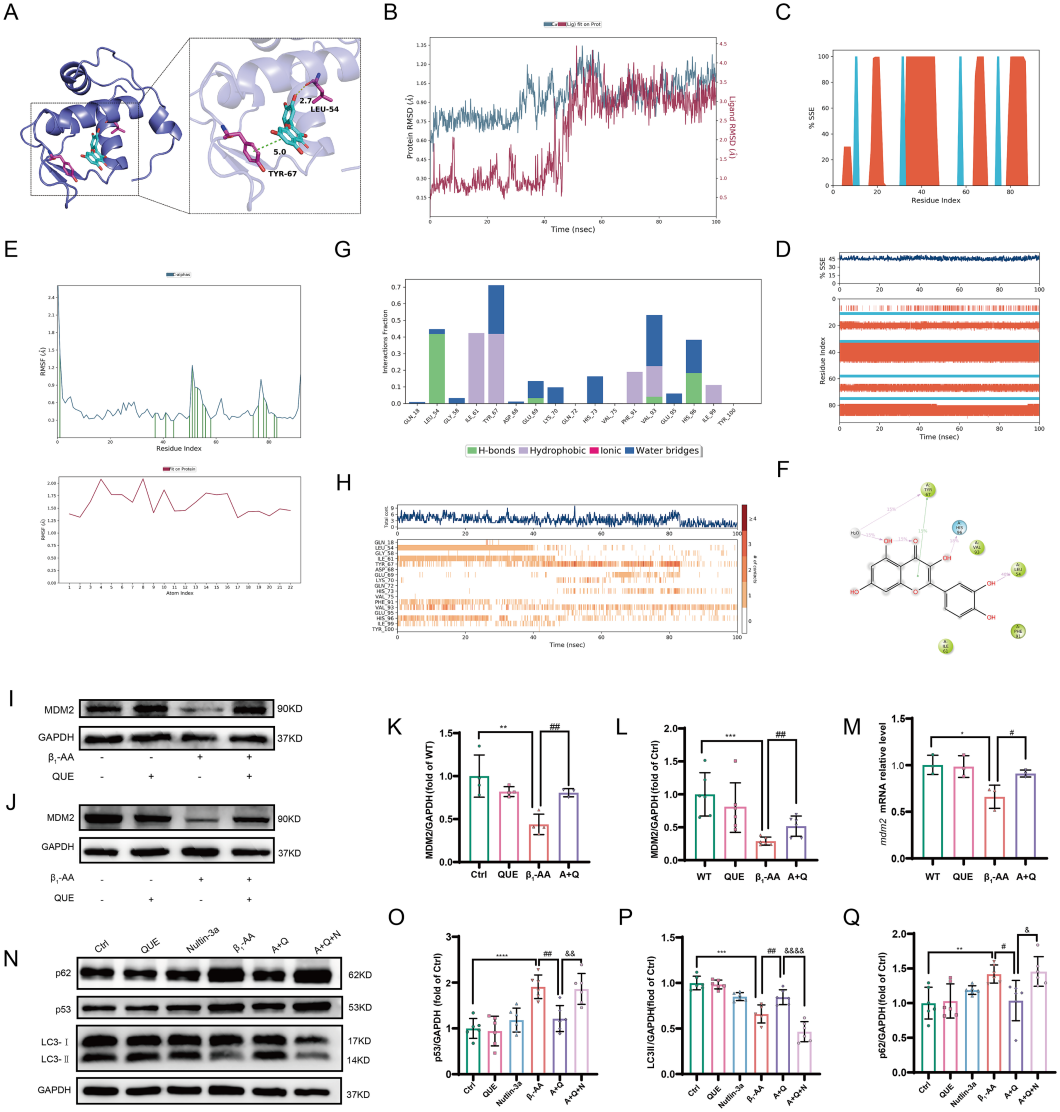
Quercetin reverses MDM2 protein downregulation in myocardial tissue induced by β_1_-AA. **(A)** Binding sites and hydrogen bonds between quercetin and MDM2. **(B)** RMSD of quercetin and MDM2. **(C)** Distribution of SSEs within MDM2, according to residue index. **(D)** Trajectory of SSE evolution and residue participation in SSEs. **(E)** RMSF of MDM2 and quercetin. **(F)** 2D structure of the interaction region. **(G)** Distribution of interaction bonds and their formation frequency. **(H)** Trajectory of the total interactions and participating amino acids per frame. **(I)** Western blot analysis of MDM2 protein levels in C57BL/6 mice actively immunized with β_1_-AR-ECII peptide segment for 4 weeks and treated with quercetin (*n* = 4). **(J)** Western blot analysis of MDM2 protein levels in H9c2 cardiomyocytes after 4 h of quercetin pre-treatment followed by 24 h of β_1_-AA treatment (*n* = 6). **(K,L)** Statistical analysis of MDM2 levels. **(M)** *MDM2* mRNA levels after 4 h of quercetin pre-treatment and 24 h of β_1_-AA treatment, as determined by RT-PCR (*n* = 3). **(N)** Western blotting analysis of LC3 and p62 protein levels after treatment with the MDM2-specific inhibitor Nutlin-3a, following quercetin reversal of β_1_-AA-induced changes (*n* = 6). **(O,P,Q)** Statistical charts for *p53*, LC3, and p62 protein expression levels. Data are presented as mean ± SD. ^**^p < 0.01, ^***^p < 0.001 vs. Ctrl; ^#^*p* < 0.05, ^##^*p* < 0.01 vs. β_1_-AA group; ^&^*p* < 0.005, ^&&^*p* < 0.01, ^&&&&^*p* < 0.0001 vs. A + Q group.

To investigate the mechanism of *p53* ubiquitination and degradation, we performed molecular docking to analyze the binding characteristics between the classical E3 ubiquitin ligase MDM2 and quercetin. The results showed that MDM2 binds to quercetin with a binding energy of < −6.9 kcal·mol^−1^ (Figure 7A), indicating that quercetin effectively targets MDM2. We then conducted molecular dynamics simulations, during which the protein RMSD remained relatively stable, eventually converging between 0.9 and 1.2 Å, while the ligand RMSD stabilized at approximately 3.2 Å after initially fluctuating. This suggested the formation of a more stable binding conformation compared to the initial state (Figure 7B). Next, we analyzed the secondary structure of MDM2. The results revealed that 36.82% of the amino acids were located in α-helical regions, while 8.60% formed β-strands (Figure 7C). Trajectory plots illustrate the composition of secondary structural elements (SSEs) and the residue distribution per frame (Figure 7D). Compared to disordered regions, these regions are predicted to contribute to more stable binding. Therefore, we evaluated the RMSF of the complex. Residues forming hydrogen bonding (green line) exhibited minimal fluctuations, confirming stable interactions in these regions (Figure 7E). A further analysis of interaction forces and their frequencies in the binding pocket revealed that the interactions were primarily mediated by hydrogen bonds, hydrophobic interactions, and water bridges (Figures 7F,G). The trajectory plots depict the total interactions and specific amino acids involved in each frame (Figure 7H). These results demonstrate that the dynamic interactions between quercetin and MDM2 remained stable over time. Intraperitoneal injection of quercetin significantly reversed the β_1_-AA-induced downregulation of MDM2 protein levels in myocardial tissue in the active immunization mouse model, providing experimental validation (Figures 7I,K). After 24 h of β_1_-AA treatment, MDM2 protein and mRNA levels were significantly reduced in H9c2 cardiomyocytes, while pre-treatment with quercetin for 4 h markedly increased MDM2 protein (Figures 7J,L) and mRNA levels (Figure 7M). Following treatment with the MDM2-specific inhibitor Nutlin-3a, the effect of quercetin on *p53* protein degradation was diminished, and its ability to reverse the β_1_-AA-induced decrease in autophagy levels was also weakened (Figures 7N–Q). These findings suggest that quercetin reversed myocardial autophagy by increasing MDM2 protein expression.

## 4 Discussion

Autophagy, vital for maintaining cellular homeostasis and stability under normal physiological conditions, efficiently transports and clears cellular debris, misfolded proteins, and toxic components through lysosomal degradation (32). In cardiac tissue, autophagy is critical for organelle renewal (33). Growing evidence suggests that autophagy is involved in the development of various cardiac diseases, indicating its potential as a therapeutic target for HF. In this study, we performed differential gene expression analysis on the myocardial tissue from HF and non-HF patients using the GEO database. Our findings revealed that DEGs were significantly enriched in autophagy-related pathways. This indicated that modulating autophagy in myocardial cells, either by enhancing or inhibiting its activity, may offer valuable therapeutic means for treating HF (34).

Immune dysfunction is one of the major causes of HF (35). Clinical studies have shown that β_1_-AA, a product of immune dysfunction, is present in the serum of patients with dilated cardiomyopathy and HF (31, 36). Overactivation of the β_1_-AR is crucial in cardiac hypertrophy and its progression to HF (37, 38). Removing β_1_-AA from patients’ blood through immunoadsorption was shown to significantly improve cardiac function (31). Our previous research confirmed that β_1_-AA activates the β_1_-AR/cAMP/PKA signaling pathway to suppress cardiomyocyte autophagy (39). Treatment with the autophagy inducer rapamycin to upregulate cardiomyocyte autophagy was shown to significantly improve β_1_-AA-induced HF. Therefore, β_1_-AA-induced suppression of cardiomyocyte autophagy is a key factor in cardiomyocyte death and cardiac dysfunction (2). However, there is currently a lack of effective drugs that can improve β_1_-AA-induced HF. Finding safe and effective drugs that upregulate cardiomyocyte autophagy is crucial for improving β_1_-AA-induced HF.

TCM has a history of over 2,000 years, and it is widely used across many countries (40). Increasing research supports the significant safety and efficacy of herbal medicine in treating HF (41). Among these, the Qiliqiangxin capsule, a traditional Chinese medicine, has shown promising results in treating HF (9). This compound herbal formula contains 11 ingredient plants: *Panax ginseng*, *Astragalus*, *Rhizoma aconiti lateralis radix preparata*, *Alisma*, *Salvia miltiorrhiza*, *Carthami flos*, *Semen descurainiae lepidii*, *Cinnamomi ramulus*, *Citri reticulatae pericarpium*, *Polygonati odorati rhizome*, and *Cortex Periplocae*. Studies have demonstrated that Qiliqiangxin capsule protected the heart in HF patients (9, 42). It was shown to improve cardiac function in mice post-myocardial infarction by enhancing mitophagy through the Pink1/Parkin signaling pathway (43), and to improve cardiac function in STZ-induced diabetic mice by promoting AGTR1/TRPV1-mediated autophagy (44). Thus, Qiliqiangxin capsule appears to enhance cardiomyocyte autophagy and slow the progression of HF through multiple targets and pathways. However, there is limited research on the precise mechanisms through which Qiliqiangxin capsule exerts its beneficial effects in HF. Network pharmacology is an emerging research method that combines systems biology, genomics, proteomics, and other disciplines to explore drug targets and their molecular mechanisms (45). This method involves virtual computation, high-throughput data analysis, and network database retrieval to elucidate the synergistic effects of multi-component, multi-channel, and multi-target interactions. Through synergistic multi-component network pharmacology and drug repurposing, precise and effective therapeutic interventions are identified, providing new means of exploring the mechanisms of TCM formulas and accelerating clinical translation. Therefore, in this study, we applied network pharmacology to systematically decipher which natural compounds in Qiliqiangxin capsule improve cardiac function by upregulating cardiomyocyte autophagy. We identified 81 gene targets related to HF, Qiliqiangxin capsule, and autophagy, suggesting that these genes may mediate the decreased autophagy in HF. We constructed an HF-target-active ingredient network, which identified quercetin as the most prominent component of Qiliqiangxin capsule in the treatment of HF.

Quercetin is a polyphenolic flavonoid widely found in fruits and vegetables (46). It is known for its diverse therapeutic effects, including neuroprotection, cardioprotection, and anti-atherosclerotic effects (47). Studies showed that quercetin prevented vascular endothelial dysfunction, reduced end-stage cardiac damage, prevented myocardial fibrosis, and regulated cardiomyocyte redox balance under high-glucose conditions. Quercetin was also found to promote desuccinylation of isocitrate dehydrogenase (IDH2) *via* SIRT5, maintain mitochondrial homeostasis, and alleviate myocardial fibrosis, ultimately reducing the risk of HF (48). Furthermore, quercetin has been shown to enhance mitophagy through the SIRT1/TMBIM6 pathway, improving mitochondrial energy metabolism and protecting cardiomyocytes (12). Quercetin regulated miR-223-3p/FOXO3 to promote autophagy and prevent isoproterenol-induced myocardial fibrosis (13). In this study, we similarly observed that quercetin effectively reversed the decline in cardiomyocyte autophagy levels induced by β_1_-AA. However, the precise mechanism through which quercetin regulates autophagy requires further exploration.

Further analysis using STRING database, PPI network, and Cytoscape revealed that *p53* is a key target of quercetin. Studies have shown that *p53* in the cytoplasm acts as an inhibitor of autophagy. In humans, mice, and nematodes, knockout or pharmacological inhibition of *p53* has been shown to induce autophagy (49). The results of this study demonstrated that β_1_-AA stimulation significantly increased *p53* protein levels in cardiomyocytes, which were effectively reduced by quercetin treatment. Further experiments showed that knockdown of *p53* markedly enhanced autophagic activity. In contrast, *p53* overexpression substantially counteracted the autophagy-restorative effects induced by quercetin. Collectively, these findings indicated that quercetin reverses β_1_-AA-induced autophagy inhibition in a *p53*-dependent manner. Moreover, we observed that while quercetin treatment significantly decreased *p53* protein levels, it did not alter *p53* mRNA levels. This suggests that quercetin does not regulate *p53* expression at the transcriptional level; instead, it may increase degradation of the *p53* protein. *p53* regulation is mainly dependent on the control of protein stability (50). To explore the mechanism of *p53* degradation, we used R packages to analyze the targets of quercetin and found significant enrichment in the ubiquitination degradation pathway. Ubiquitination, a crucial post-translational modification, is closely related to cardiovascular diseases (51). We then used Co-IP to assess *p53* ubiquitination levels and found that quercetin indeed reversed the decline in *p53* ubiquitination levels induced by β_1_-AA, thus normalizing *p53* protein expression. To further investigate the mechanism of *p53* ubiquitination and degradation, we performed molecular docking between *p53* and the classical E3 ubiquitin ligase MDM2. The results indicated a binding affinity of <−6.9 kcal·mol^−1^. Experimental validation confirmed that β_1_-AA reduces MDM2 protein levels, while quercetin restored MDM2 expression. Furthermore, the MDM2-specific inhibitor Nutlin-3a diminished the effect of quercetin on *p53* protein degradation and weakened the ability of quercetin to reverse the β_1_-AA-induced decline in autophagy levels. This confirmed that quercetin increased MDM2 expression in cardiomyocytes, which mediated *p53* ubiquitination and degradation, thus reversing the β_1_-AA-induced decline in autophagy levels.

## 5 Conclusion

**Figure 8:**
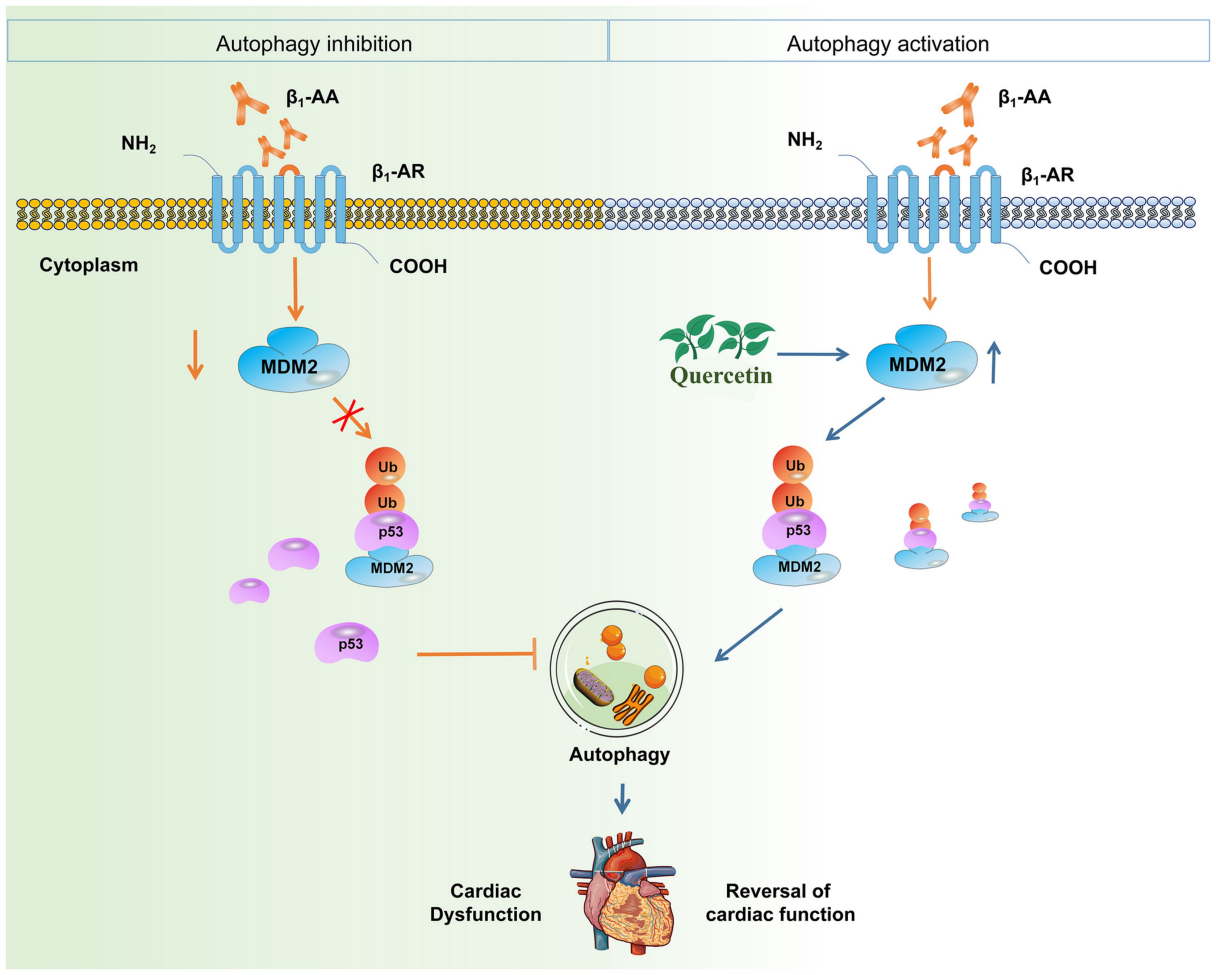
Quercetin alleviates β_1_-AA-induced HF by enhancing autophagy via the MDM2/*p53* pathway. This study demonstrates that quercetin counteracts β_1_-AA-induced HF. The core mechanism involves quercetin upregulating MDM2 expression, which promotes the ubiquitination and degradation of *p53*. This subsequently relieves the inhibitory effect of *p53* on cardiomyocyte autophagy, ultimately restoring autophagic flux and conferring cardioprotection.

In conclusion, our study unveils a novel cardioprotective mechanism of quercetin against β_1_-AA-induced HF. We definitively established that quercetin alleviates HF by restoring impaired myocardial autophagy. The core finding is that quercetin achieves this effect by targeting the *p53* protein. Specifically, we identified that quercetin promotes the MDM2-mediated ubiquitination and degradation of *p53*, which reverses the autophagy suppression caused by β_1_-AA (Figure 8). These findings not only elucidate a new molecular pathway through which quercetin exerts its therapeutic effects but also provide a solid experimental foundation for targeting the *p53*-MDM2-autophagy axis as a promising strategy for treating autoimmune-related heart failure.

## Data Availability

Publicly available datasets were analyzed in this study. This data can be found here: https://www.ncbi.nlm.nih.gov/geo/, accession numbers GSE57338, GSE5406, and GSE3586.

## Footnotes

^1^ https://old.tcmsp-e.com/index.php

^2^ https://db.idrblab.net/ttd/

^3^ https://disgenet.cn

^4^ https://go.drugbank.com/

^5^ https://string-db.org/

^6^ https://www.rcsb.org/

## Glossary

β_1_-AA: β_1_-adrenergic receptor autoantibody
β_1_-AR: β_1_-adrenergic receptor
SA: Streptavidin
ECII: Second extracellular loop
TCMSP: Traditional Chinese Medicine Systems Pharmacology Database
OB: Oral bioavailability
DL: Druglikeness
GEO: Gene expression omnibus
DEGs: Differentially expressed genes
BSA: Bovine serum albumin
DMEM: Dulbecco’s modified eagle medium
PBS: phosphate-buffered saline
DAPI: 4′,6-diamidino-2-phenylindole
QUE: Quercetin
p53: Tumor protein P53
MDM2: Murine double minute2
Co-IP: Co-immunoprecipitation
FS: Fractional shortening
EF: Ejection fraction
LVIDs: Left ventricular end-systolic diameter
LVIDd: Left ventricular end-diastolic diameter
ESV: End-systolic volume
EDV: End-diastolic volume
