# Supplementary material for: Quercetin reverses β1-adrenoceptor autoantibody-induced heart failure by promoting MDM2-mediated ubiquitination and degradation of p53 in cardiomyocytes
Source: Front Nutr. 2025 Oct 16;12:1674507. doi: 10.3389/fnut.2025.1674507 (PMC12573670; doi:10.3389/fnut.2025.1674507)
Supplement: Supplementary file 1 [file Data_Sheet_1.doc]

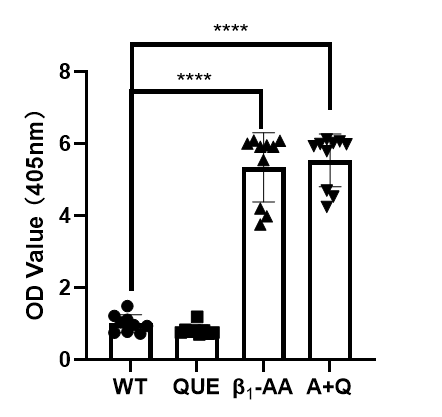


Figure S1 OD values of serum β_1_-AAs in mice after active immunization for 4 weeks. *** *P*< 0.001 vs. control.


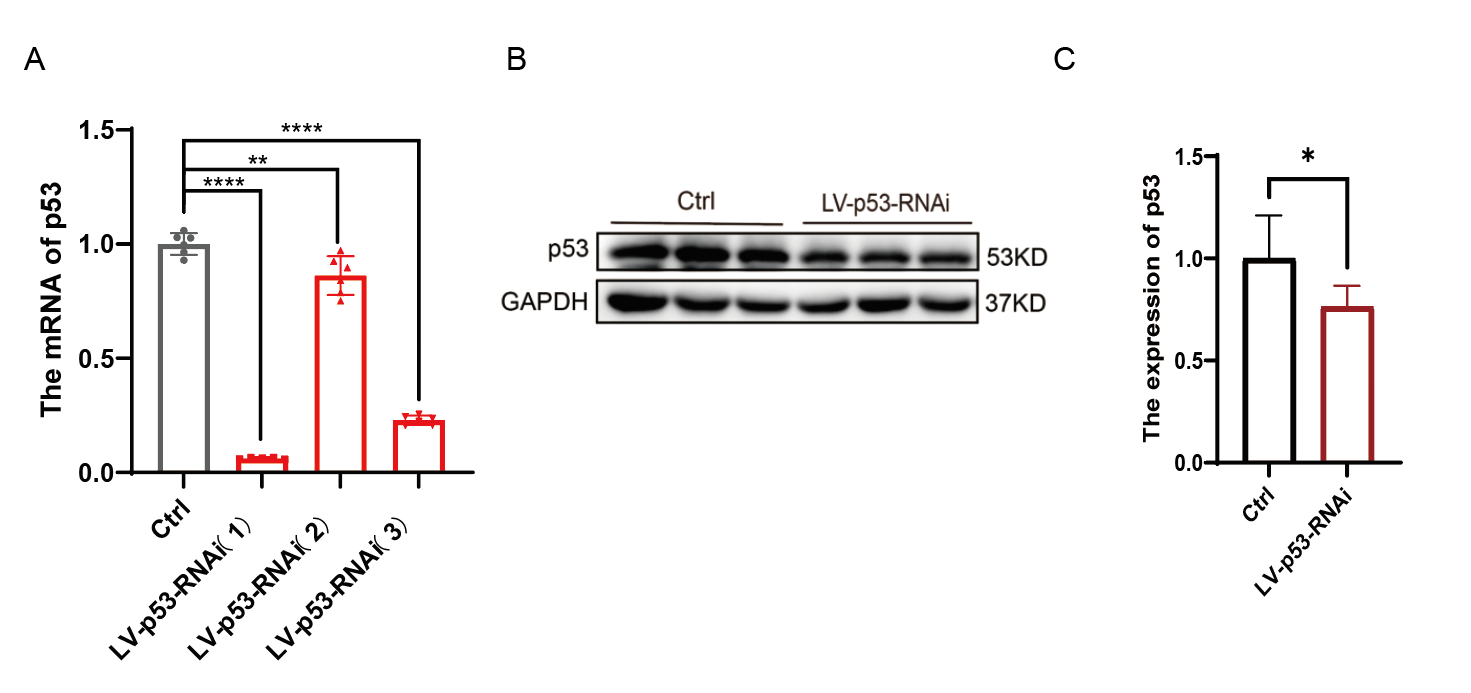


Figure S2. Lentivirus-mediated p53 knockdown effective sequence screening. (A) Verify the knockdown efficiency of p53 using three lentiviral sequences by RT-PCR. (B) (C) Verify the knockdown efficiency of p53 with lentiviral sequence 1 using Western Blot (Data are presented as means ± SD, **P* < 0.05,***P* < 0.001 ,**** *P* < 0.0001 vs. Ctrl).


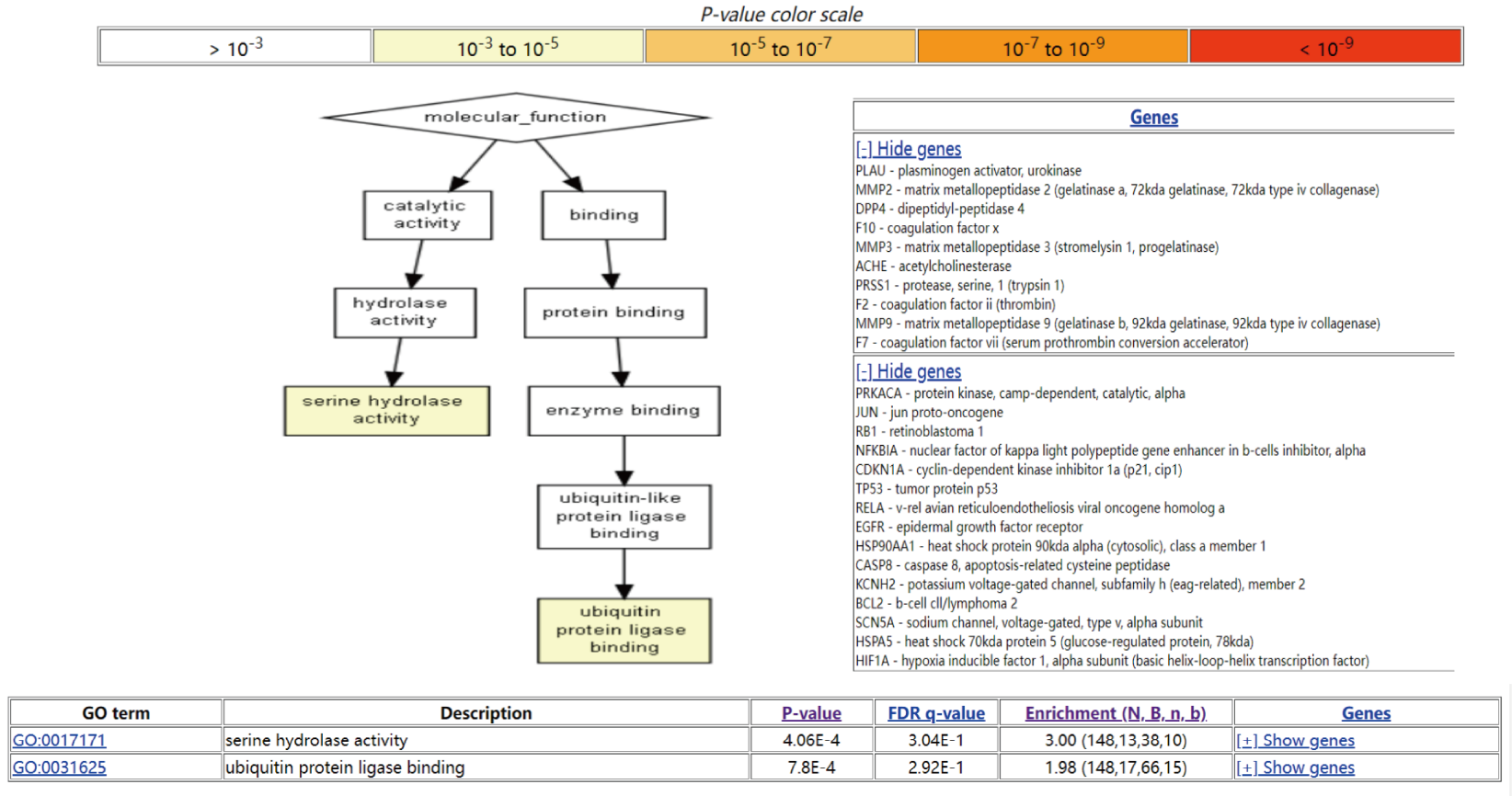
Figure S3. GO functional enrichment analysis of quercetin target genes was performed using the GOrilla database. Yellow indicates the significantly enriched ubiquitination pathway.
